# The Assassination of Anton Cermak, Mayor of Chicago: A Review of His Postinjury Medical Care

**DOI:** 10.1055/s-0040-1709459

**Published:** 2020-06-16

**Authors:** Theodore N. Pappas

**Affiliations:** 1 Department of Surgery, Duke University School of Medicine, Durham, North Carolina

**Keywords:** Cermak, assassination, colitis

## Abstract

Anton Cermak was the mayor of Chicago in the 1930s. He was injured by an assassin's bullet intended for the president-elect, Franklin Delano Roosevelt. Cermak was taken to a local hospital, treated nonoperatively for his injuries, and initially improved. Cermak's condition deteriorated on the sixth day postinjury, with symptoms that his doctors described as colitis. He died of sepsis on the 19th day after the shooting, and his autopsy revealed a perforated colon causing peritonitis. This study will review Cermak's clinical course and autopsy findings to determine if he died of his gunshot wound or if he died of complications of toxic colitis.

On February 15, 1933, Franklin Delano Roosevelt was in Miami returning from a fishing trip in the Bahamas. As he passed through Miami, Roosevelt planned to give a short speech and meet, informally, with Chicago Mayor Anton Cermak. Roosevelt stopped at the Biscayne Bay Park after 9 pm and gave his 1-minute speech while sitting in his car. After the speech, Cermak approached Roosevelt's car and sat with the president-elect discussing the financial future of Chicago. As Cermak was leaving Roosevelt's car, several shots were fired by a lone gunman attempting to assassinate Roosevelt. Roosevelt was uninjured, but Cermak and four other bystanders were stuck by errant bullets. 1 Cermak was taken to Jackson Memorial Hospital where he died 19 days later. 2 This study will review Cermak's health prior to the shooting and also the care he received during his stay at Jackson Memorial.

## Background

**Fig. 1:**
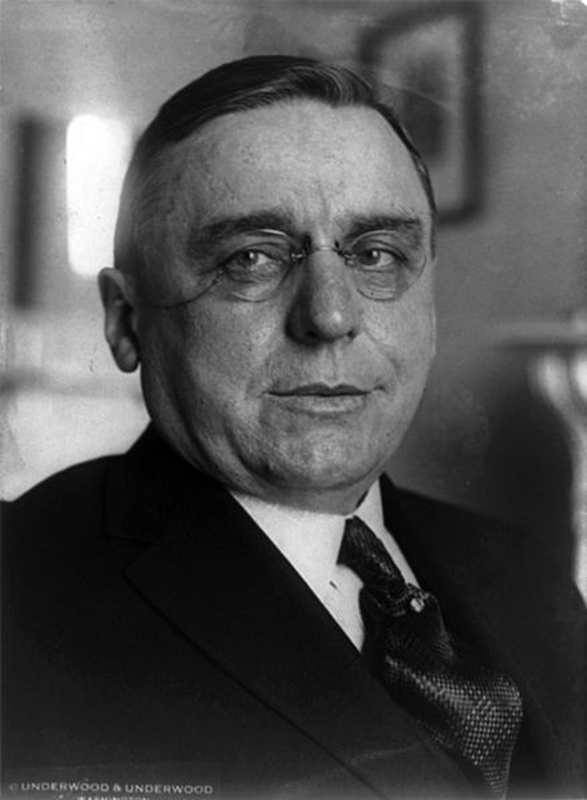
Anton Cermak (1933).

**Fig. 2:**
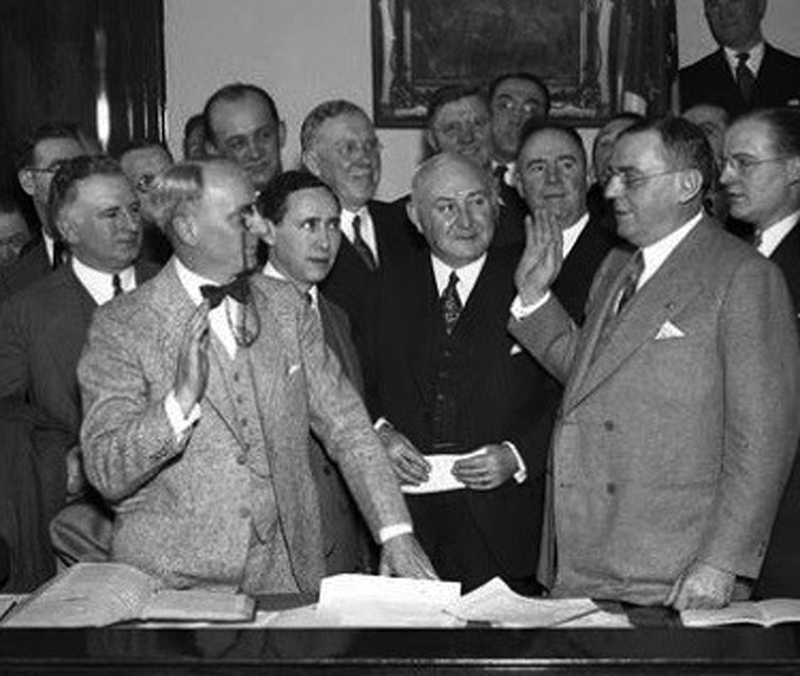
Cermak sworn into office as the mayor of Chicago (April 7, 1931).

Anton Cermak was a Czech immigrant that rose to power in Chicago politics in the environment of the major financial depression of the early 1930s. ( Fig. 1 ) He was born in 1873, came to the United States with his parents when he was 1 year old, and moved to Chicago at the age of 16 years. He began his political career as a member of the Democratic Party in 1902 when he was elected to the Illinois House of Representatives. He defeated a Republican incumbent and became the mayor of Chicago in 1931 3 ( Fig. 2 ).

## Cermak's History of Intestinal Disease

Cermak's political career was challenged by frequent severe intestinal symptoms, which were called colitis. The first public report concerning Cermak's health was in 1918, when newspapers reported that he was ill with “a severe cold.” 4 The press attributed his illness to “overwork.” On August 18, 1925, Cermak became sick while fishing near Hayward, WI. He was admitted to a local hospital due to “severe cold with intestinal trouble.” 5 He was transferred to St. Anthony's Hospital in Chicago the following day, where his temperature was 104F. He was diagnosed with “influenzal infection of the intestine” and cared for by Dr. Frank J Jirka, Cermak's son-in-law, and Karl Meyer, MD, a noted Chicago surgeon. 6 Two days later, his condition worsened as he continued to spike temperatures, but his symptoms eventually resolved and he was discharged without complication. 7 8

Cermak was diagnosed again with “intestinal inflammation” in February of 1929 and spent time recovering in Miami Beach Florida. 9 By the middle of April, he had not improved and traveled to Baltimore, MD, to be admitted to the Johns Hopkins Hospital. 10

Cermak did well for nearly a year but was sick again in the spring of 1930 and 1931 when he returned to Miami each time for rest and recuperation. 11 12 After being elected the mayor in April of 1931, 13 Cermak's illness returned and his son-in-law, Dr. Jirka, encouraged him to take a significant break from his work to avoid a “serious breakdown.” 14

Cermak's recovery was again short-lived because he was back in Florida on January 11, 1932, this time resting at a relative's home at Miami Beach. 15 He was suffering from a “severe cold and intestinal inflammation” and “confined to bed.” 16 17

Cermak was admitted again to St Anthony's Hospital in Chicago on July 6, 1932, with severe fatigue. He was described by Dr. Jirka as “rundown” and was discharged 10 days later, and he recovered from his “attack of indigestion.” 18 19 20

## Meeting Roosevelt in Miami

**Fig. 3:**
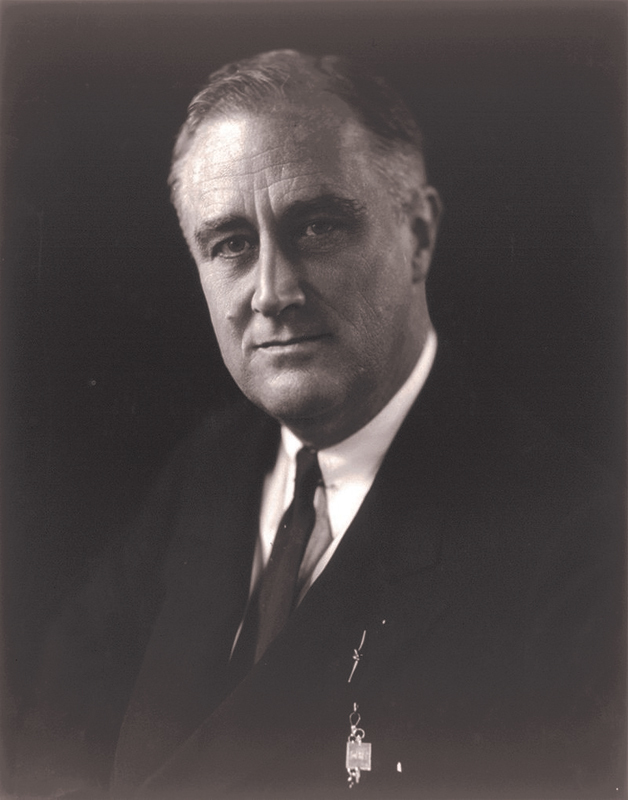
Franklin Delano Roosevelt, Presidential portrait (1933).

When Cermak became the mayor, Chicago was in significant financial distress and in need of federal support. Cermak planned to meet with Roosevelt to discuss federal programs that might support Chicago's needs. 21 In February of 1933, Cermak agreed to meet James Farley (chair of the Democrat National Party), in Miami, to discuss Roosevelt's support of Cermak's efforts in Chicago. The mayor also planned to meet Roosevelt in Miami to confirm Farley's promises. 20 Roosevelt was returning from a Caribbean fishing trip in early February and was passing through Miami for a brief stop as he traveled back to New York. 22 23 Roosevelt arrived in Miami on the evening of February 15 and was to be driven by his security team to Biscayne Bay Park where he would make a brief speech and then proceed by train to New York. Given the limited time that Roosevelt was to be in Miami, it was suggested that Cermak meet Roosevelt just after the short speech ( Fig. 3 ).

Roosevelt arrived at the park after 9 pm and his car stopped in front of the large crowd that had been waiting for the president-elect to deliver his speech. Roosevelt sat on the top of the back seat of his convertible to give his 1-minute speech around 9:40 p.m . After the speech, Cermak, who was sitting with other dignitaries just steps away from the convertible, moved to the running board to shake hands and speak with the president-elect. After speaking with Roosevelt, as Cermak moved away from the convertible, Giuseppe Zangara fired five shots in an attempt to assassinate Roosevelt. 24

## Assassin

**Fig. 4:**
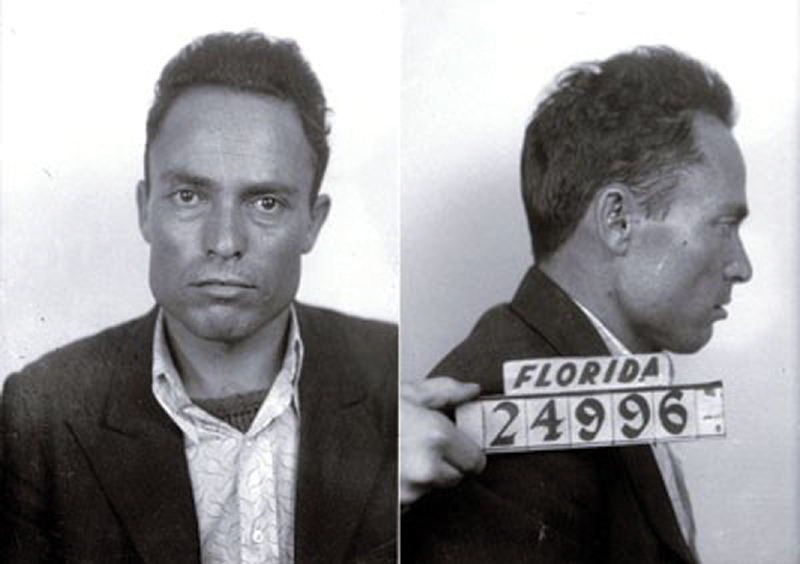
Giuseppe Zangara, mugshot Florida Department of Corrections.

Giuseppe Zangara, an often unemployed bricklayer, was born in Italy, came to the United States in 1923, and became a naturalized citizen in 1929. He was sitting in the fifth or six throw of seats, 20 feet from Roosevelt when he fired his revolver. 25 Lillian Cross and Tom Armour were private citizens sitting next to Zangara, who disrupted his aim and were given credit for saving the future president's life. 26 Five individuals were hit by bullets including Mayor Cermak, but Roosevelt was not injured. Zangara ( Fig. 4 ) was immediately apprehended and later told police that he wanted to kill kings, presidents, and all capitalists. 27 Cermak fell after being stuck in the right flank by a single bullet. He was able to stand with assistance and helped into Roosevelt's car, which quickly drove 20 city blocks to Jackson Memorial Hospital.

## Jackson Memorial Hospital

When Cermak got to the hospital, he was noted to have an entrance wound on the right chest, just below the tip of the scapula. The physicians stated that the bullet had traversed the right lung, the right diaphragm, and the liver. The trajectory of the bullet was posterior and downward, and it was detected by X-ray in the 11th thoracic vertebra. By 2 a.m., the physicians taking care of Cermak issued a statement that the mayor had an expected 50% mortality but they were not recommending immediate surgery. He was afebrile with a heart rate of 88 beats per minute and was breathing comfortably at 24 breaths per minute. 28

At approximately 20 hours after the shooting, Cermak was described by his physicians as “very satisfactory.” Again his vital signs were stable (pulse of 88 beats per minute, respiratory rate of 22 breaths per minute, temperature of 99°F), and his pain from the bullet injury was diminishing. Plans for an operation were again delayed since the mayor was stable and the bullet location was not causing harm. 29 They thought the kidney was spared and again suggested that the diaphragm and the liver had been traversed. 30

Two physicians, well known to Cermak, joined the team of doctors who were taking care of the mayor. The first was Dr. Frank J. Jirka, who had recently been appointed the Director of the Illinois Department of Public Health. The second was Dr. Karl Meyer, the head of the Cook County Hospital and a surgeon with extensive experience with abdominal operations. 30 31 32

At 10:30 p.m. on February 16, the mayor had stable vital signs (pulse rate of 96 beats per minute, temperature of 99.6°F, and respiratory rate of 20 breaths per minute) despite the fact that he had developed heart block on his EKG (electrocardiogram). The physician team worried about “weakening of the heart” and the possibility that the mayor might develop pneumonia. 33 His condition improved on February 17, when vital signs were stable, and he was able to speak to his family. The surgeons confirmed that the bullet went through the tip of the right lung in its path from lateral to medial, but they were convinced that Cermak would recover barring complications such as pleural effusion or empyema. 34 35

By February 18, the mayor had improved sufficiently to sit up in bed and talk to reporters for the first time since the shooting. His doctors including, his son-in-law, continue to be cautious, stressing that complications could still cause a setback. By all accounts, Cermak appeared to be starting to emerge from danger. 36 February 19 and 20 were similar days, marked only by a low-grade fever of 100F each day. 37 38

### Cermak Develops Colitis

Cermak's condition started to deteriorate on February 21. In the evening, he developed a fever of 101F, rigors, and abdominal pain associated with a heart rate of 108 beats per minute and respiratory rate of 30 breaths per minute. The physicians stated that the cause of decline was “colitis.” 39 By the following day, the abdominal pain from the colitis was somewhat improved, but his loose stools continued. His heart rate was 130 beats per minute, and he was described as exhausted. 40

During the night of February 22 and the following morning, Cermak became hemodynamically unstable, with hypotension and decreased urine output, and was presumed to be in “shock.” His blood pressure was sufficiently low and therefore his heart rate could not be measured for three minutes. He was given intravenous dextrose (1,500 mL of a 10% glucose solution) and caffeine as a “stimulant” to raise his blood pressure. During this crisis, the doctors briefly thought Cermak had died. He responded to this resuscitation, and by the afternoon of February 23, his vital signs and renal function had stabilized and improved. 41 The physicians continued to worry about his respiratory function and planned to use an oxygen tent if his respiratory rate increased further. 42

February 24 was a stable day, but on February 25, the mayor had worsening cardiopulmonary function. 43 The physicians no longer predicted survival but simply said Cermak would “live through the night.” He was placed in an oxygen tent and received his first blood transfusion along with his daily glucose infusion. His gastrointestinal symptoms persisted, and the physicians continued to call this condition colitis. Blood donors were sought since daily transfusions were anticipated. 44

On February 26, the mayor was diagnosed with an infection in his right lung, which was confirmed by X-ray in the setting of decreased breath sounds on auscultation. According to his physicians, it was unclear if this represented a posttraumatic abscess or pneumonia. 45 Cermak was stable on February 27, his breathing slightly less labored, and he was temporarily removed from his oxygen tent. 46 His downhill course continued on February 28 when his infiltrate on chest X-ray doubled in size. When the mayor's respiratory rate reached 40 breaths per minute and he was gasping for air, he was placed back in the oxygen tent. 47 Despite some encouraging words from his physicians, there was clear evidence that Cermak deteriorated on March 1 and 2. He was moved from the oxygen tent to an oxygen room to guarantee his high-flow oxygen. 48 The colitis was still present, his respiratory rate continued at 30 breaths per minute, and hiccups started on March 2. 49

**Fig. 5:**
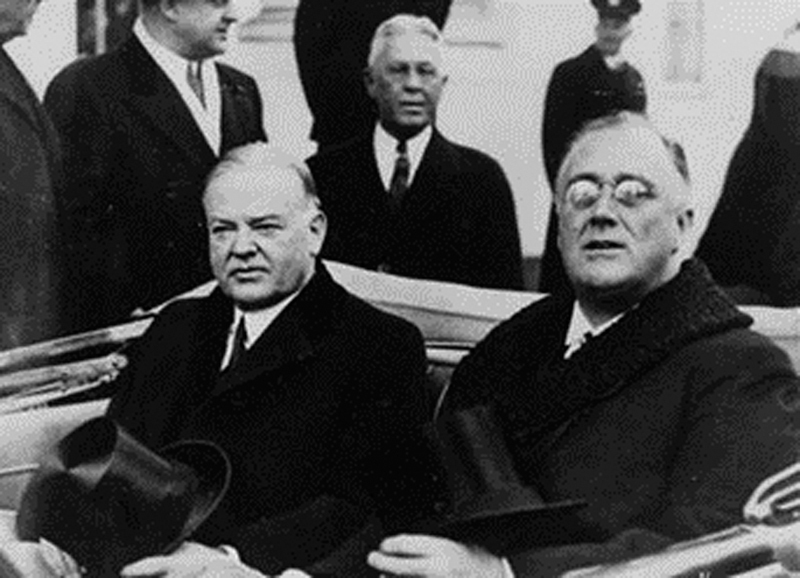
Franklin Delano Roosevelt and Herbert Hoover riding to Roosevelt's first inaugural address (March 4, 1933).

On March 3, Cermak's abdominal pain and distension worsened and was accompanied by persistent shortness of breath and right shoulder pain. The physicians were concerned about empyema or right subphrenic abscess. He was taken to the operating room on March 4 for a needle aspiration of a right chest plural effusion. The aspirate was smelling foul; therefore, a chest tube was placed, which drained bloody serous fluid described as a “gangrenous process” in the right lung. 50 51 On the same day in Washington, DC, Roosevelt was inaugurated at the 32nd President of the United States 52 ( Fig. 5 ).

Cermak spent the March 5 getting transfusions and intravenous fluids for worsening heart rate and low blood pressure, but by 12:05 a.m. on March 6, he was in a coma. 53 Attempts at resuscitation included neoarsphenamine (an arsenic preparation used to fight gangrene). 54 The mayor died at 6:57 a.m. on the morning of March 6. 55

## Aftermath

Zangara was originally indicted for attempted murder, but the charge was changed to murder following the death of Cermak. After a speedy trial, where Zangara admitted that he was trying to kill Roosevelt, he was convicted and sentenced to death. Zangara was executed by electric chair in the Florida State prison, Raiford, FL, on March 20, 1933. 56

The other four individuals injured on February 15 recovered from their wounds. Mabel Gill was shot in the abdomen, required a laparotomy for her wounds, and was discharged from the hospital on March 23, 1933, after a complicated course. 57 William Sinnott, a former New York police officer, and 22-year-old Russell Caldwell both recovered from bullet injuries to the head. Margaret Kruis, a 23-year- old dancer, had a minor injury to her hand. 58 59

## Autopsy

The Cermak autopsy was attended by nine doctors. The conclusion by the doctors was that the gunshot wound initiated a downhill course that triggered severe colitis. They stated that the bullet had injured the lung and diaphragm, which caused hemorrhage and cardiac failure. The colitis was triggered by the systemic stress of the bullet injury. The colitis progressed to ulcerative and then toxic colitis, resulting in right colon perforation and gangrene above and below the right diaphragm. 60

The summary of the autopsy was presented at the trial of Zangara. The physicians made it clear that the gunshot wound was the cause of death because it initiated a cascade of events culminating in colitis, colon perforation, and peritonitis. There was concern that the assassin would attempt to say that the bullet injury was nonlethal and the doctor's mismanagement caused the eventual death. This was the attempted defense that Charles J. Guiteau used when he was tried for the assassination of President Garfield. 61 Despite the apparent consensus among the physicians, as shown by their signatures on the autopsy report, there was disagreement as to the importance of the colitis in the mayor's course. Several newspaper reports appeared in the month following Cermak's death, which shed a different light on the cause and effect of the gunshot wound. On March 30, Dr. Meyer stated that the mayor would certainly have healed his chest wound had he not developed colitis. 62 This opinion was corroborated on April 1 by Dr. Frederick Tice, a physician attending the autopsy, when he stated that “The bullet wound was not directly responsible” for Cermak's death. 63 Both physicians claimed that the mayor was “rundown” in an overall weakened state, which contributed to his difficulty in fighting these complications. The state's attorney, N. Vernon Hawthorne, who tried the case that led to the execution of Zangara, took issue with these opinions. He sent a public letter to Dr. Meyer, attached a copy of the autopsy, and reminded the surgeon that the stated cause of death was the bullet wound. 64 Meyer attempted to backtrack his comments under pressure from the state's attorney, but he still maintained that the final cause of death was from complications of the colitis. 65

## Analysis of Cermak's Care

Cermak was shot with one bullet that entered his back just below his right scapula and lodged in the body of T11 vertebra. There is evidence that it traversed the right pleural cavity, the right lung, and the right diaphragm before it lodged in the spine. Although the autopsy and news reports state that the lung was punctured, traversed, and collapsed, there is no mention of a right-sided chest tube early in the management. Given that chest tubes were commonplace in the 1930s, it is very likely that his pneumothorax was small and stable and therefore not treated with a tube. 66 He had a chest tube place just before his death for an empyema.

Cermak stabilized quickly after the shooting and steadily improved over these first 5 days. The clinical course is consistent with the described injury. A gunshot wound to the right chest, right diaphragm, and dome of the liver would not require intervention if the pneumothorax was small and the bleeding minimal. After 5 days of improvement, the mayor developed gastrointestinal symptoms consistent with his history of colitis. The mayor had fever, abdominal pain, rigors, and diarrhea, and the autopsy described ulcerative and gangrenous colitis. Given the mayor's long history of colitis, it is likely that he developed an exacerbation of existing ulcerative colitis as he recovered from the gunshot wound.

The gastrointestinal symptoms worsened during his hospital course, similar to toxic colitis. Late in his hospital course, the mayor developed hiccups and right shoulder pain likely as he perforated his right colon and formed a subphrenic abscess. If his bullet wound injured the right diaphragm, a subphrenic abscess could easily necessitate into the right chest, resulting in the need for drainage of the foul-smelling pleural fluid just prior to his death. His autopsy findings align with his clinical course, suggesting that he developed toxic colitis and perforation of the right colon with subphrenic abscess. Two of the physicians attending the autopsy explicitly state that the colitis, colon perforation, and peritonitis caused the mayor to die, not the gunshot wounds.

### Management of Ulcerative Colitis in the 1930s

The medical management of ulcerative colitis was largely supportive in the 1930s. Patients with severe diarrhea were often dehydrated and benefited from intravenous hydration, as did Cermak. Otherwise, there was no proven medical treatment of ulcerative colitis at the time of Cermak's illness. 67 The use of sulfonamides was evaluated for patients with ulcerative colitis in 1942, and corticosteroids were not used to treat ulcerative colitis until 1950. 68 69 Once a patient developed toxic colitis, the mortality was extremely high, and emergency total abdominal colectomy was not considered. Occasionally, surgeons used diverting ileostomy as urgent treatment in the toxic patient and would follow with colectomy only if the patient stabilized. 70

### Alternate Hypothesis to Explain Cermak's Course

It has been suggested that the real reason for his colon perforation was a missed bullet injury to the right colon. In his book titled “The Five Weeks of Giuseppe Zangara,” author Blaise Picchi asked several Miami surgeons to review Cermak's hospital course as chronicled by the newspapers of the day. After their review, the surgeons hypothesized that when the bullet passed through the right diaphragm, it injured the right colon, leading to subphrenic abscess, peritonitis, and death. 71 Although this explanation is possible, it ignores many of the details of the 19-day clinical course. This alternate explanation implies that Cermak survived a bullet wound to the colon without surgery for 19 days in the preantibiotic era. In addition, it is unlikely that a bullet injury to the colon would present with diarrhea. Picchi's Miami surgeons also failed to review Cermak's long history of colitis requiring several hospital admissions prior to the shooting.

## Summary

Anton Cermak sustained a gunshot wound to the chest from an assassination attempt originally meant for the president-elect, Franklin Delano Roosevelt. Cermak was taken to Jackson Memorial Hospital in Miami where he died 19 days later. He was treated nonoperatively for a penetrating chest wound. As he was recovering from a nonlethal gunshot wound, he developed severe ulcerative colitis, a disease he had been hospitalized for many times in the past. He died of complications of toxic colitis, which included right colon perforation, right subphrenic abscess, and peritonitis.
